# Notes and News

**Published:** 1902-08-23

**Authors:** 


### Notes and News.

The King's gift of Osborne House and estate to the nation is quite unhampered with any provision of occasional accommodation for Royal personages. As a Convalescent Home for naval and military officers it possesses the greatest advantages, having a private landing stage on the Solent, and being within easy reach by water from both the naval and and military hospitals of Haslar and Netley and from the Port of Southampton.

Her Royal Highness the Princess Frederica (Baroness von Pawel-Rammingen) has consented to become Patron of the Central London Ophthalmic Hospital.

There has been much activity in the construction of hospital accommodation lately. The Marquis of Bute has laid the foundation-stone of the new Seamen's Hospital at Cardiff. At Meltham Moor the foundation-stone of an Isolation Hospital for the Colne and Holme Valleys has been laid by Mr. Thomas Mallinson, J.P.; at Pontypool Mrs. J. C. Hanbury has performed the same office for a General Hospital, and so has also Mr. John Musgrave for a new Workhouse Hospital for Whitehaven. At the Glasgow Royal Infirmary a new electrical pavilion has been opened.

One feature in connection with the new electrical department at the Glasgow Royal Infirmary is a Wimshurst machine, one of the largest in existence, for the production of electricity. This machine was built by Lord Blythswood in his laboratory at Renfrew, and was a gift by his lordship to the hospital. The X-ray department has been refurnished with the newest switchboards, coils, and operating tables. In addition, the best apparatus [illegible] localising the presence of foreign substances in [illegible] body have been provided. The older and well-[illegible]n apparatus for constant and interrupted [illegible]ents, used for diagnosis and treatment, have [illegible]een replaced by those of the most modern kind. A large electric magnet for the extraction of metallic foreign bodies has been specially designed, and the general department includes electric drills, saws, trephines, and electric probes for detecting the presence of bullets, etc.

An International Congress on the Care of the Insane will be held on September 1st at Antwerp. The boarding out system will especially be under discussion, as adopted at Gheel and Lierneux.

A new laboratory is being fitted up at the Cancer Hospital, Fulham Road, and Mr. Plimmer, who has been pathologist to the institution for many years, will be appointed ditector, and a pathologist has been elected to work under him in the investigations which will be much increased owing to the facilities which will be afforded in the new laboratory.

There are upwards of 380 cases of cholera in Egypt. In Cairo there are about 30, the mortality being large; there are about 40 to 50 cases in each at Beirut, Gizeh, Enbabeh, Mineh and Bebris, and about 20 at Alexandria. The cases have numbered upwards of 2,000 since the commencement of the epidemic, and two-thirds of these have been fatal.

The deliberations of the Select Committee appointed to inquire into the ventilation of the House of Commons do not appear to promise any very drastic measures being resorted to. The most definite statement so far is that the air in the building, from a bacteriological point of view, is, on the whole, good. This conclusion will lead many who suffer from the atmosphere in the House to hope that the test of common sense and practical experience will have due weight when recommendations are to be made, for, microbes or no microbes, the air is frequently oppressive and exhausting to those who have to breathe it for any length of time.

The punishment of one month's imprisonment inflicted upon a man who went about amongst the public when suffering from smallpox may seem somewhat severe to some, in the face of his defence, which was that he was not responsible for his actions at the time. But the measure should be regarded as none too drastic as an object lesson. Exposing unsuspecting persons to the danger of small-pox infection cannot be too vigorously punished, when the consequences are considered. Conscientious objections to vaccination may be permitted, especially when small-pox is not rife, but any objection to isolation must be totally disregarded to the extent of punishment if evaded, or the law is useless to protect the public.

Mr. H. D. Kerr, who describes himself as Hon. Treasurer, V.C.C., sends us the following interesting record:—"The success of vegetarian athletes in the last Continental long-distance walk (from Dresden to Berlin, 124½ miles) should result in special consideration being given to the value of the "fleshless" diet for feats of endurance. Out of 32 starters, 18 were vegetarians; and of the 13 who finished the course, 10 (including the first 6) were vegetarians. The winner, Karl Mann, a member of the Vegetarian Cycling Club of London, occupied 27 hours 13 mins., or, deducting time lost in the Government inspection at various points on the route, 26 hours 52 mins.—an average of nearly five miles an hour throughout! At 75 kilometres he was inside world's track record, despite the difficulties of the road and the fact that he encountered almost continuous thunderstorms. Such a performance is phenomenal."

